# Actomyosin contractility requirements and reciprocal cell–tissue mechanics for cancer cell invasion through collagen-based channels

**DOI:** 10.1140/epje/s10189-022-00182-6

**Published:** 2022-05-16

**Authors:** Lianne Beunk, Gert-Jan Bakker, Diede van Ens, Jeroen Bugter, Floris Gal, Martin Svoren, Peter Friedl, Katarina Wolf

**Affiliations:** 1 Department of Cell Biology, Radboud University Medical Center, 6525 GA Nijmegen, The Netherlands; 2 David H. Koch Center for Applied Research of Genitourinary Cancers, Department of Genitourinary Medical Oncology, The University of Texas MD Anderson Cancer Center, Houston, TX USA; 3 Cancer Genomics Center, Utrecht, The Netherlands

## Abstract

**Abstract:**

The interstitial tumor microenvironment is composed of heterogeneously organized collagen-rich porous networks as well as channel-like structures and interfaces which provide both barriers and guidance for invading cells. Tumor cells invading 3D random porous collagen networks depend upon actomyosin contractility to deform and translocate the nucleus, whereas Rho/Rho-associated kinase-dependent contractility is largely dispensable for migration in stiff capillary-like confining microtracks. To investigate whether this dichotomy of actomyosin contractility dependence also applies to physiological, deformable linear collagen environments, we developed nearly barrier-free collagen-scaffold microtracks of varying cross section using two-photon laser ablation. Both very narrow and wide tracks supported single-cell migration by either outward pushing of collagen up to four times when tracks were narrow, or cell pulling on collagen walls down to 50% of the original diameter by traction forces of up to 40 nN when tracks were wide, resulting in track widths optimized to single-cell diameter. Targeting actomyosin contractility by synthetic inhibitors increased cell elongation and nuclear shape change in narrow tracks and abolished cell-mediated deformation of both wide and narrow tracks. Accordingly, migration speeds in all channel widths reduced, with migration rates of around 45-65% of the original speed persisting. Together, the data suggest that cells engage actomyosin contraction to reciprocally adjust both own morphology and linear track width to optimal size for effective cellular locomotion.

**Graphic abstract:**

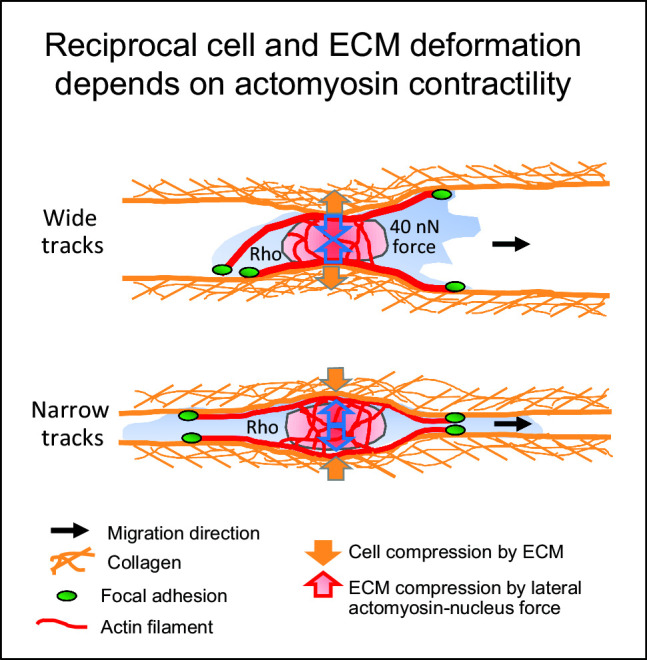

**Supplementary Information:**

The online version contains supplementary material available at 10.1140/epje/s10189-022-00182-6.

## Introduction

Tumor progression is accompanied by the spread of tumor cells to adjacent and distant tissues through invasion and metastasis and, consequently, reduced prognosis on patients’ life span [1, 2]. Tumor-surrounding tissues range from extracellular matrix (ECM)-rich connective tissues and stiffened desmoplastic tumor stroma to nearby fat and muscle layers [3, 4]. Invading cancer cells are therefore confronted with ECM that, besides a range of stiffness values, consists of different topologies, including discontinuous pores bordered by fibrillar collagen or continuous channel-like tracks of linear or nonlinear shape [5]. *In vivo*, the degree of confinement can vary considerably, with spaces in randomly organized collagen-rich networks ranging from 1 to 20 $\upmu$m [6] and in linear-organized topologies in or near perimuscular or perineural tissues from 2 to 30 $\upmu$m in diameter [7]. Whether these different geometries impose different mechanical and molecular requirements on invading cells remains unclear.

Mesenchymal cell invasion into complex substrates potentially providing physical constraints has been conceptualized as a multi-step process. Actin-mediated cell polarization and protrusion of the leading edge are followed by integrin-mediated substrate attachment for transient anchorage and actomyosin contraction of the cell body, with the latter two jointly generating the traction force that enables cells to pull on substrate, accompanied by focalized substrate proteolysis to widen ECM gaps, and finally followed by de-adhesion at, and forward-slipping of, the cell rear [8–11]. Cell locomotion also includes the actomyosin contraction- and tubulin-dependent forward motion of the cell nucleus, the cell’s stiffest and largest organelle [12–15]. Actomyosin contractility depends on myosin II intercalation between actin filaments leading to their displacement and overall stress fiber shortening [16]. Myosin-II activity is regulated by the small Rho GTPase RhoA that acts through its effector protein Rho-associated coiled coil-containing kinase (ROCK) enabling the phosphorylation of the myosin II light chain [17]. RhoA/ROCK signaling, by regulating cell body contraction and adhesion strength, determines traction force and migration efficacy [9, 18, 19]. Indeed, interference with the Rho/ROCK axis reduces tumor cell migration speed *in vitro*, with outreach to reduced metastasis formation *in vivo * [20–23].

Actomyosin contractility function on migration may depend on the topology and the degree of cell confinement by the extracellular environment. Cells are confined when they are embedded in extracellular substrate that causes the adaptation of a polarized cell’s typical cross section to smaller areas [22]. Accordingly, interference with contractility reduced migration efficacy in soft confining collagen hydrogels *in vitro* [22, 23],  but also in wider linear soft collagen-based microtracks of 200 $$\upmu $$m$$^{2}$$ cross section [24]. Conversely, interfering with cell contraction in confining (30 $$\upmu $$m$$^{2})$$ linear stiff polydimethylsiloxane (PDMS) microchannels left cell speed unaltered [25]. Thus, while Rho/ROCK-dependent actomyosin contractility impacts cell migration in collagen-based discontinuous three-dimensional (3D) networks or larger tracks, in stiff confinement contractility-independent migration modes exist. Whether confined linear *in vivo*-like interstitial environments can support Rho/ROCK-independent migration remains unclear.

We here developed an assay of engineered microtracks of various defined cross sections in a collagen lattice ranging from 7 - 460 $\upmu$m$^{2}$ and probed the necessity of actomyosin contractility for cell migration in dependence of lateral confinement. Different from previously used PDMS-based approaches, the 3D collagen track structure was deformable, hence allowing for reciprocal adjustment of cell-channel width by pulling on and pushing of walls by the moving cells. Using pharmacological interference with Rho/ROCK signaling, we found that migration speed was reduced in all track diameters, while ECM deformation was abolished. The data suggest that in pliable linear environments, actomyosin contractility is required for ECM pulling in large tracks or pushing in confining tracks to deform, and hereby accommodate, track diameters to cellular and nuclear shape to enable high-speed migration.

## Material and methods

**Reagents and antibodies:** The following dyes and antibodies were used for microscopy: Alexa Fluor 568-conjugated phalloidin (#A12380, ThermoFisher), DAPI (Sigma) and polyclonal affinity-purified rabbit anti-COL1 $\frac{3}{4}$ C short antibody (Immuno-Globe) directed against the C-terminal cleavage neo-epitope of collagen type I [26]. For Western blotting, polyclonal rabbit antihuman phospho-myosin light chain 2 antibody (Thr18/Ser19; #3674, Cell Signalling Technology), polyclonal rabbit antihuman myosin light chain 2 antibody (#3672, Cell Signalling Technology) and Odyssey system-specific antibodies goat anti-rabbit 608 and goat anti-rabbit 800 (both Li-Cor Bioscience) were used. For functional studies, the following small synthetic drugs were used: ROCK-inhibitor Y-27632 (H$_2$O-dissolved, at indicated concentrations; Santa-Cruz); Myosin II-inhibitor blebbistatin (DMSO-dissolved, at indicated concentrations; Sigma); and MMP-inhibitor GM6001 (DMSO-dissolved, 5 $\upmu$M; Calbiochem).

**Cell culture:** The following cell lines were used: wildtype human fibrosarcoma HT1080 cells (ACC315; DSMZ Braunschweig), or HT1080 cells expressing either cytoplasmic TagRFP in combination with nuclear histone-2B (H2B)-coupled eGFP, cytoplasmic eGFP in combination with H2B-coupled mCherry, or H2B-coupled mCherry alone. Expression of fluorescent markers was induced by transduction with a lentiviral vector and did not affect migration kinetics (data not shown). All cells were cultured (37°C, 10% CO$_{2}$, humidified atmosphere) in Dulbecco’s modified Eagle’s medium (DMEM; Invitrogen) supplemented with 10% fetal calf serum (FCS; Sigma), penicillin (100 U/ml; PAA), streptomycin (100 $\upmu$g/ml; Invitrogen), L-glutamine (2 mM; Lonza) and sodium pyruvate (1 mM; Gibco).

**Collagen microtrack migration assay:** Fibrillar collagen lattices (end concentration of 6 mg/ml) were generated by preparing a mixture of acid collagen type I solution (rat tail; Corning) supplemented with 10x Minimum Essential Serum (Sigma) and 1N NaOH with a resulting pH of 7.4. 100-200 $\upmu$l of this mix was added to a self-constructed wax–glass [22] or silicon–glass chamber of 1-2 mm thickness. Fibrillar collagen gels were formed upon polymerization at 37°C for 15 minutes after which culture medium was added. Chambers were sealed and stored for up to 24 hours before laser ablation. Tracks of indicated cross sections and a length of approximately 1 mm were dissected from the collagen network using a Ti-sapphire laser (Chameleon Ultra II, Coherent) at 830 nm, a scanning frequency of 50 Hz, a pixel dwell time of 21 $\upmu$s and pixel size of 0.32 $\upmu$m, and a laser power of approximately 300 mW under the objective (Nikon 2.5x 1.1 NA water objective) of a multiphoton microscope setup (TriMScope II; LaVision BioTec). Cell-free microtracks were imaged using forward detection of second harmonic generation signal generated by excitation at 830 nm, a line frequency of 800 Hz, and a laser power of 35 mW under the objective. Within 24 hours after track generation, chambers were opened, cell culture medium removed, HT1080 cells seeded on top of the track entrances, and chambers filled up with culture medium. Before re-sealing the chamber, chambers were cultured in an upright position for at least 1 hour to allow cell attachment to collagen and equilibration of the medium. Collagen lattices and culture medium were supplemented with GM6001 to enable cell migration in guiding tracks bordered by intact collagen and, where indicated, with Y-27632 or blebbistatin, and subjected to life microscopy for 24 hours.

**Time-lapse microscopy and quantification of cell migration:** Cells were monitored using a Zeiss Axiovert 200M microscope (plan-NEOFLUAR 10x/0.3 NA air objective) equipped with a Moticam-pro 2850 CCD camera, an Okolab stage incubator (37°C, 10% CO$$_{2})$$ and run by Micromanager 1.4 software for 22-25 h with 4-min frame intervals. Migration speed from individual cells was quantified using manual computer-assisted tracking of xy paths with 4-min time intervals [Autozell software, Centre for Computing and Communication Technologies (TZI), University of Bremen, Germany]. The mean speed per cell in the tracks was calculated by dividing path length by time and therefore included also the ‘stop’ phases.

**Confocal fluorescence and reflection microscopy:** Cell–collagen samples were fixed with 4% PB-buffered paraformaldehyde (PFA), washed, stored at 4°C and stained with fluorescent dyes as indicated. Imaging of fixed samples was performed using a FV1000 confocal microscope (Olympus, 40x/0.8 NA water objective). For confocal time-lapse microscopy, an SP5 confocal laser scanning microscope system (Leica, 40x/0.5 NA objective) was utilized.

**Scanning electron microscopy:** To visualize the microtracks in the collagen lattice at single fibril resolution, scanning electron microscopy (SEM; Sigma 300, Zeiss) was utilized. As preparation, cell-free samples were fixed for 1 hour at room temperature (RT) with 2% glutaraldehyde (Merck) in 0.1 M cacodylate buffer, followed by two washes in the same buffer. 1% osmium tetroxide (EMS) in 0.1 M cacodylate buffer was used as a secondary fixative with incubation for 1 hour at RT. Samples were processed by dehydration through a graded series of ethanol followed by critical point-drying using CO$_{2}$ and then sputtered with a conductive metal and imaged.

**SDS-Page and Western blotting:** Sub-confluent adherent cells were washed and directly exposed to Laemmli lysis buffer on ice. Where indicated, cells were pre-treated with inhibitors of actomyosin contractility for 1 hour. Lysates were harvested, boiled for 10 minutes at 95°C and stored at -20°C. Samples were separated by 15% SDS-PAGE electrophoresis, transferred onto polyvinylidene fluoride (PVDF) membranes, blocked using Li-Cor blocking buffer (Li-Cor Biosciences), incubated with primary anti-pMLC antibody at 4°C overnight and a secondary antibody for 1 hour, and fluorescence was detected using the Odyssey system (Li-Cor Biosciences). To strip blots, western blot stripping buffer (Restore Plus, ThermoScientific; for wet PVDF membranes) or harsh stripping buffer (62,5mM Tris pH 6.8, 0,5% SDS, 0,7% $\beta$-mercaptoethanol; for methanol-reactivated dried PVDF membranes) was used. After confirmation of complete stripping by fluorescence detection, blots were blocked again with Li-Cor blocking buffer before a second staining with MLC-specific and secondary antibody and fluorescent bands were detected. Fluorescent intensities were quantified using Li-Cor Biosciences ImageStudio Lite Version 5.2.

**Gel contraction assay:** Collagen type I lattices (300 $\upmu$l; 1,7 mg/ml; rat tail) containing 420.000 cells were polymerized in triplicates in a 48-well cell culture plate, loosened from the well, and culture medium was added. Where indicated, Y-27632 or blebbistatin was added to both collagen and supernatant. After incubating plates for 48 hours, decrease in collagen gel surface was monitored at a dissection microscope equipped with a camera.

**Cell viability assay:** Cells were cultured in a 48-well culture plate with indicated inhibitor concentrations. After 24 hours, the culture medium potentially containing dead cells was collected, and adherent cells detached and mixed with their original supernatant. The cell suspension was incubated with 1 $\upmu$l of propidium iodide for 1 minute and measured at a FACScalibur machine (BD Biosciences) for propidium iodide-positive cells using the 585/40 filter. Data were analyzed using the FlowJo software in which the gating was set based on the control samples.

**Proliferation assay:** Cells were cultured and treated with indicated inhibitor concentrations in triplicates in plastic flat-bottom 96-well culture plates for 24 hours, and subsequently fixed with 4% PFA. Fixed samples were stained with DAPI and the entire well imaged using epifluorescence microscopy combined with automated multi-position image acquisition and stitching (Leica DMI6000B, 10x/0.9 NA water objective). Numbers of cell nuclei per condition were determined by automated DAPI counting as explained in the section ‘Image analysis.’

**Image analysis:** Image processing and quantification were performed by Fiji ImageJ (1.52n; National Institutes of Health) [27]. Images were cropped, rotated, manually adjusted for contrast and brightness and displayed in virtual colors. Images were reconstructed as maximum intensity projections from all fluorescence z-scans and 1 reflection scan. Areas of contracted gels were measured manually using the polygon selection, and track width and minor nuclear diameter using the straight line function. For quantification of proliferation, tile scans were stitched into a single image, thresholded (Otsu white), processed using watershed function and the number of nuclei counted in an automated fashion. The nuclear irregularity index (NII) [28, 29] was determined in an automated fashion, with mal-segmented nuclei being excluded manually. To determine cell length and nuclear position over time, cells were tracked on three points, leading edge, nucleus center and cell rear using Manual Tracking plugin in Fiji Image J, and the mean was formed. For quantification of proteolysis, the mean gray value of the COL1 ¾signal per image was determined, subtracted from background signal and divided by the number of nuclei in an image. For track deformation analysis, a z-plane intersecting the complete track within a region of interest was selected and stabilized over time [30]. Fluorescence channels were spatially median-filtered (2 pixels), and the reflection channel was spatiotemporally mean-filtered (xy: 1 pixel, time: 2 frames). Kymographs were generated using the multi-kymograph analysis function in Fiji. The reflectance channel was median-filtered (4 pixels) prior to kymograph generation. Last, the manual tracking Fiji plugin was used to track the collagen speckle pattern over time. Manual tracks were imported into MATLAB (Version 2018b), and a custom script (to be provided upon request) was used to calculate the strain and force over time.

**Force calculation:** The quantification of force on the track wall over time was based on a simplified linear relation between cell-induced *Stress* and *Strain *within the collagen matrix ($E=$*Stress/Strain*), where (1) *Strain* $=$ relative deformation of the collagen with respect to time point 0, (2) *Stress *$=$ cell-induced force *F* divided by cell–collagen contact area (a square of 20 $\upmu$m x 20 $\upmu$m$^{2}$, thus 400 $\upmu$m$^{2}$ area, was assumed, providing a rough estimate), and (3) Youngs modulus $E_{collagen\, [6mg/ml]} =$340 Pa (interpolated value from a range of determined stiffness values from rat tail collagen preparations of 3-7 mg/ml [31]).

**Statistics:** Statistical analysis was performed by Kruskal–Wallis test with Dunn’s multiple comparisons tests to detect differences between multiple groups with non-Gaussian distribution, or the Mann–Whitney test to detect differences between two groups with non-Gaussian distribution using GraphPad Prism (version 8) software.

## Results

**Generation of collagen-ablated microtracks**

To investigate tumor cell invasion into linear channel-like structures of very small, intermediate and large cross section relative to cell size, and in order to achieve standardized conditions, we adapted a pre-existing assay of two-photon laser-generated microtracks in collagen lattices [32]. Linear tracks were cut into a polymerized high-density 3D collagen lattice using two-photon laser ablation with an opening to the collagen–medium interface (Fig. 1A). To match the range of pre-existing tissue track widths observed *in vivo* [7], various track diameters were generated, with diameters between 2.6 and 21 $\upmu$m and rectangular cross sections ranging from 7 to 460 $\upmu$m$^{2}$ size (Fig. 1B, C). Because of the small difference in pre-set versus measured diameters, 2 $\upmu$m pre-set tracks were in the following named ‘narrow’ tracks, and 20 $\upmu$m pre-set tracks were named ‘wide’ tracks (Fig. 1C). The ablation yielded smooth cutting edges of collagen fibrils without damage to the surrounding collagen (Fig. 1D). These barrier-free tracks of various cross sections in the same collagen lattice were used for multiplexed side-by-side analyses of cancer cell invasion.

**Fig. 4:**
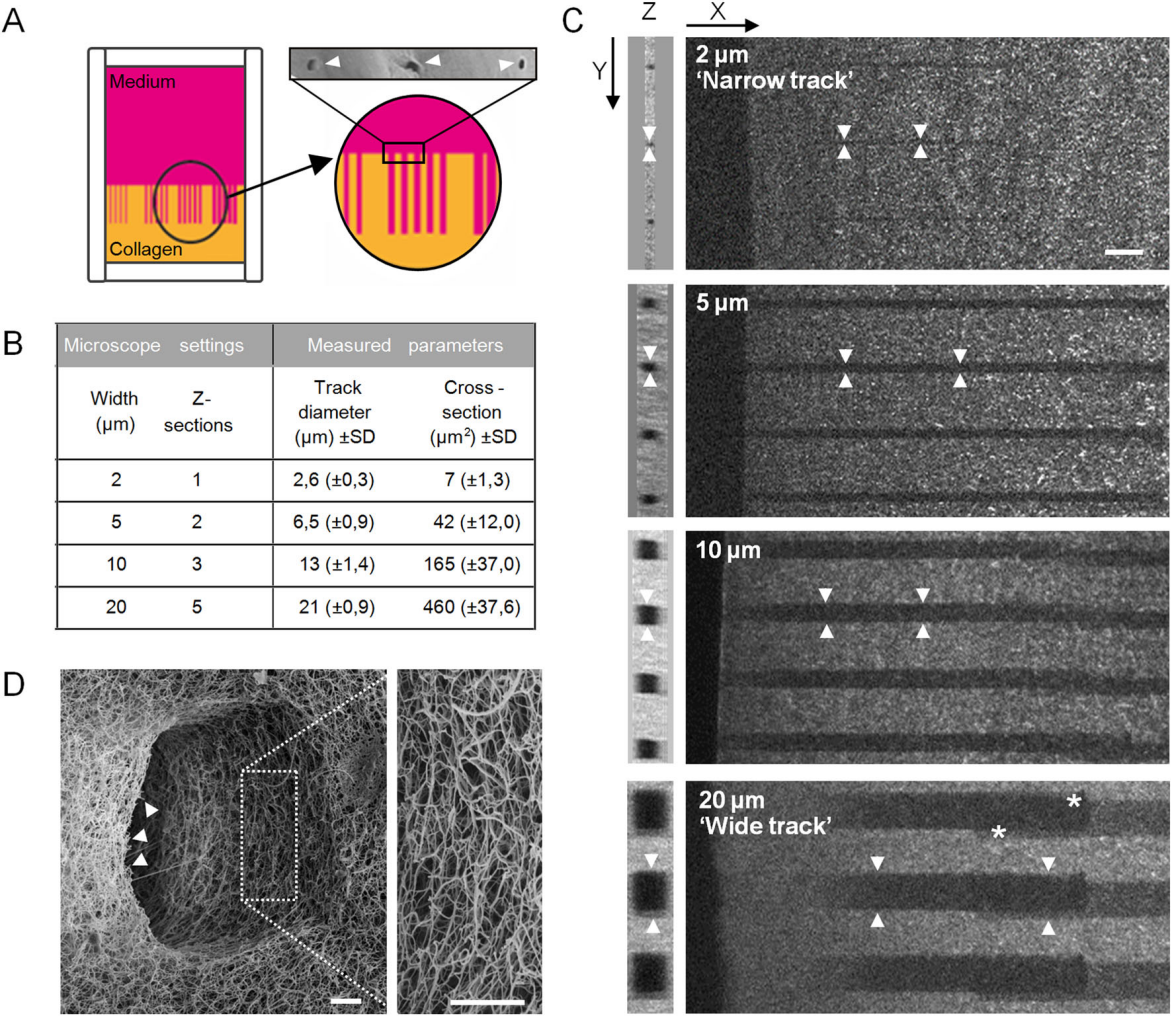
Setup of a collagen model with microtracks of varying cross-sections. **A** Schematics depicting the setup of a collagen lattice within a 3D chamber from which microtracks of varying cross-sections were laser-ablated. Top right, entrances of three microtrcks into collagen (arrowheads), imaged by scanning electron microscopy. **B** Overview of used microscope settings during laser ablation and indicated output parameters measured from negative reflection signal by confocal reflection microscopy. **C** Microtracks (arrowheads) of indicated microscope-set diameters after laser ablation. Images were generated from PFA-fixed collagen samples by second harmonic generation, with loss of signal indicating ablated regions. Drifting of the sample during laser ablation sometimes resulted in slightly shifted ablated areas (asterisks). **D** Entrance of 10 $\upmu \hbox {m}$ wide microtrack imaged by scanning electron microscopy. Arrowheads indicate cleanly cut fibers. Scale bars: 20 $\mu$m (**C**); 2.5 $\mu$m (**D**)

**Fig. 5:**
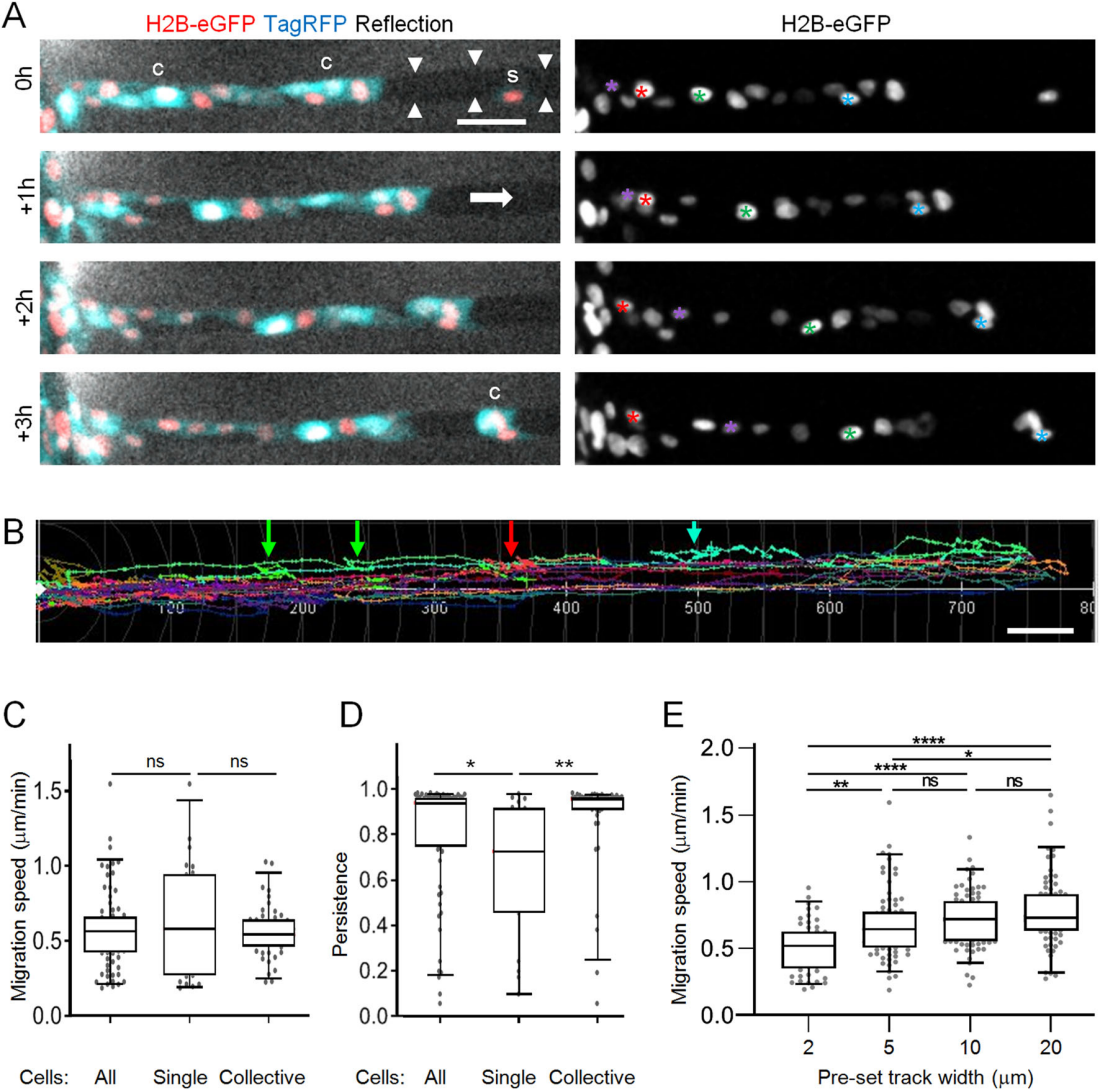
Characterization of HT1080 cell migration in microtracks. **A** All cells migrated within collagen-ablated wide track with a general migration direction from left to right (see white arrow). Images of cells with indicated nuclear and cytoplasmic labels within track (arrowheads) at different time points over 3 hours. Differently colored asterisks indicate individual nuclei. S, single moving cell; C, collectively moving cells. **B** Trajectories of cells in wide tracks over 24 h. Colorful arrows point to regions of respectively colored trajectories where cells changed migration direction. **C** Average speed per cell migrating as single cell or within a collective as indicated in (**A**). **D** Persistance of cells migrating as single cells or within a collective. Persistance was calculated as track length divided by the beeline. **E** Mean migration speed of individual cells over 6–22 hours in tracks of varying diameters. Data represent 53–80 cells per condition (*N* = 3), and are also shown in Fig. 4C). Horizontal. lines and boxes and whiskers show the medians, 25th/75th, and 5th/95th percentile. Dots show all individual measurements outside of the 25th to 75th percentile. Mann-Whitney test. Ns, not significant; *, *p*-value $< 0.05$; **, *p*-value $< 0.01$; ****, *p*-value $< 0.0001$. All scale bars: 50 $\mu$m

**Fibrosarcoma cell migration in microtracks in single and collective mode**

To enable tumor cell migration entry intro microtracks, we overlaid mesenchymal HT1080 fibrosarcoma cells onto the top of the collagen, thus providing direct access to the entrance of the pre-existing microtracks. To minimize collagen degradation by HT1080 cell-expressing matrix metalloproteinases (MMPs), including MT1-MMP or MMP-2 [26, 33, 34], all experiments were performed in the presence of the broad-spectrum MMP-inhibitor GM6001, in order to maintain an intact microtrack architecture for cell guidance (Figure S1). After entering the wide tracks, migrating HT1080 cells started to follow the pre-existing channel structure individually, but then merged into multi-cellular clusters, (Fig. 2A, B; Supplementary Movie 1). The migration efficacy of both single or collectively moving cells was unaltered, and all cells predominantly moved in forward direction, but single cells had a higher tendency to occasionally also move backward, thus moved with less persistence than grouped cells (Fig. 2B-D). In the different channel widths, cells migrated with average speeds ranging from 0.5 $$\upmu $$m/min in narrow (7 $$\upmu $$m$$^{2})$$ to 0.7 $$\upmu $$m/min in wide (460 $$\upmu $$m$$^{2})$$ tracks (Fig. 2E). The average speed range maintained within all tracks exceeded the average migration speed observed for HT1080 cells in randomly polymerized fibrillar collagen (0.3 $$\upmu $$m/min) [22]. Thus, the pre-existing microtrack architecture supported efficient fibrosarcoma cell migration, alone or in groups, in tracks of all tested diameters.

**Modulation of track diameters by fibrosarcoma cell-mediated pushing and pulling during migration**

Migrating cells not only sense, integrate and adapt to signals from the environment, but reciprocally respond to, and thereby change, properties of the invaded microenvironment [5]. Consistently, both outward and inward deformation of the track boundary by migrating cells was observed. Single cells and, to greater extent, file-like cell clusters migrating through confining microtracks had a larger diameter than the pre-generated diameter of narrow tracks (Fig. 3A, left). As a result, cells migrating in narrow tracks pushed track boundaries outward to diameters 3-8 times larger than the original diameter, in agreement with previously published data [32], and with residual widening persisting after cells had migrated through these tracks (Fig. 3B, left). In wide channels, when transmigrated by either single cells touching one side of the wall only, or by collectively migrating cells, the track diameters remained similar to the pre-existing sizes (Fig. 3A, B; each right). In addition, increased reflection signal was observed along the walls of wide tracks after cell transmigration, implying traction force-mediated collagen reorganization during migration (Fig. 3A, right). However, when single cells attached to both sides of the walls, they dynamically narrowed track diameters similar to the size of the cell body, which resulted in smaller diameters than the original track widths, and partially persisted after cell transmigration (Fig. 3B, C; Supplementary Movie 1). When measured on four different example cells, track diameters narrowed from 22 $\upmu$m original size down to 17 $\upmu$m and up to 11 $\upmu$m, thus reduced in size by 23–50% (Fig. 3D). Thus, to generate cell size-matching track diameters associated with effective migration, cells push ECM away in confining spaces or pull on wide boundaries [35].

**Fig. 6:**
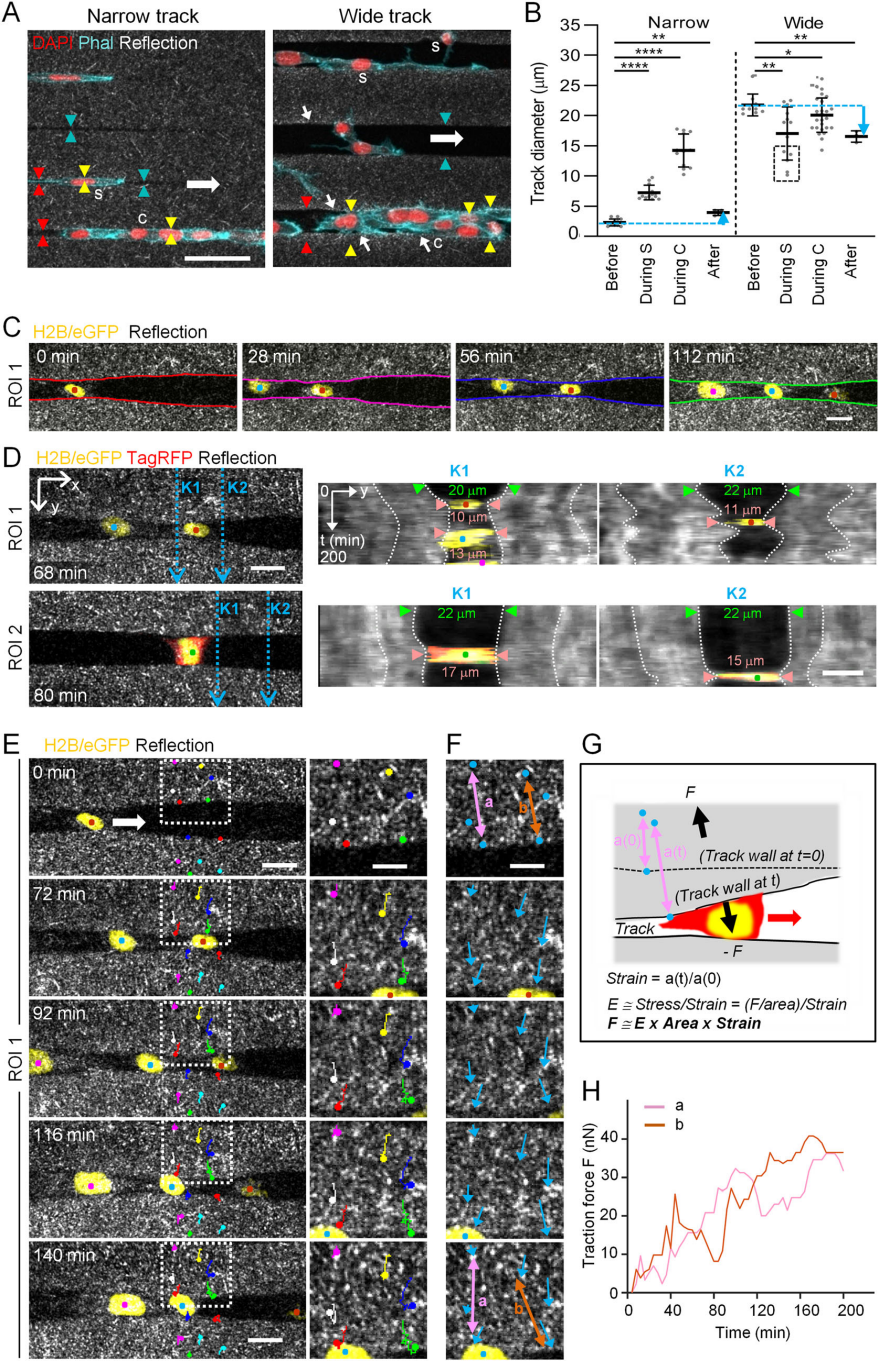
Quantification of HT1080 cell-induced collagen deformation during microtrack migration. All cells migrated within $\blacktriangleright$ collagen-ablated wide tracks with a general migration direction from left to right. **(A)** Single cells (s) and cell clusters (c) migrating in tracks of indicated widths. Arrowheads, visualization of track boundaries before (cyan), during (yellow) and after (red) cell passage; note the equal distance between arrowheads along the track length. Small white arrows indicate collagen densification. **(B)** Track widths before, during (’During S’, during single cell transmigration; ’During C’, during collective cell transmigration) or after cell passage of narrow or wide tracks. Data represent 3-27 measurements (dots) per condition (*N*=2). Solid line with whiskers, mean and SD. Cyan dotted lines, initial track diameter. Cyan arrow indicates difference between track diameter before and after cell transmigration. Black dotted rectangle, deformed track diameters by single cells attaching to both track walls. Kruskal-Wallis test with Dunn’s multiple comparisons test. *, *p* -value < 0.05; **, *p* -value < 0.01; ****, *p*-value < 0.0001. (C-F) 3D timelapse confocal microscopy of cell-induced track deformation, where 80 $\mu$m stacks with 5 mm step sizes were taken at 4-min time intervals. Each of the three different cell nuclei is color-coded by dots throughout these panels. **(C)** Imaging sequence taken from a plane in the middle of a track at indicated time points, corresponding to the upper part (ROI1) of Movie 1. Color-coded lines indicate track wall deformation. **(D)** Kymograph analysis from two image sequences from the upper (ROI1) and lower (ROI2) part of Movie 1. From the x-positions K1 and K2 of both ROIs (left, blue dotted arrows), track deformation over time is shown (K1, middle; K2, right). White dotted lines show deformation of the collagen as a function of time (vertical axis). **(E)** Tracking of the local collagen speckle pattern (colored dots and lines) over time. Dotted rectangle depicts region of zoom-in on the right, demonstrating that collagen displacement is increasing towards the track edge (compare long red and green with short pink and yellow colored trajectories). **(F)** Strain analysis by cell-induced change of collagen speckle distance over time. Blue arrows, magnitude and direction of collagen displacement with respect to the first time point (0). Colored double arrows a and b in upper and lower image indicate increased distance between track edge and speckle-like positions within the collagen after cell passage. **(G)** Cartoon depicting collagen deformation (strain) by transmigrating cell and calculation principle of cell-derived traction force onto collagen leading to track deformation. The depicted formulas are applied for the calculation of traction force F. **(H)** Quantification of F on the track wall over time. Scale bars: 50 $\mu$m (A), 20 $\mu$m (C; D and E, left), 10 $\mu$m (D and E, right; F)

To quantify the forces that migrating cells apply on their environment, we focused on cells pulling onto the collagen walls of wide tracks. The emerging strain field was characterized by tracking speckles within the collagen lattice, obtaining trajectories that delivered displacement vectors within the collagen matrix upon traction by migrating cells (Fig. 3E, F; Supplementary Movie 2). From these, we could calculate the strain and, estimating a collagen elasticity of 340 Pa and a cell–collagen contact area of 400 $\upmu$m$^{2}$, determine the emerging traction forces per cell of up to approximately 40 nN (Fig. 3G, H). This force is sufficient to lead to the narrowing of wide tracks and, although not tested here, might be as well sufficient to push collagen outward.

**Migration efficacy and modulation of microtrack width are mediated by actomyosin contractility**

The modulation of track diameter by cell-mediated pushing and pulling forces during migration implies the involvement of actomyosin contractility. To test whether actomyosin activity is required for migration and track deformation, we initially measured MLC phosphorylation and collagen contraction after titrating the small synthetic inhibitors Y-27632 and blebbistatin. Increasing concentrations of Y-27632 gradually decreased the amount of pMLC (Supplementary Figs. 2A and 3), similar to other cells and models [22, 36]. Blebbistatin caused a dose-dependent increase in the pMLC/MLC ratio by nearly 400% (Supplementary Figs. 2B and 3), consistent with the compensatory activation of the Rho pathway in response to myosin-II inhibition [36, 37]. Accordingly, both ROCK or myosin II inhibition compromised HT1080-induced contraction of collagen lattices in a dose-dependent manner (Fig. 4A). We further pursued treatment with a 20 $\upmu$ M concentration for both inhibitors, which inhibited collagen contraction near-maximally, while 40 $\upmu$ M did not significantly increase this effect further. To rule out toxicity effects, we confirmed that HT1080 cells remained viable in the presence of each inhibitor at the selected dose (Supplementary Fig. 2C). In addition, Y-27632 did not compromise cell proliferation, whereas blebbistatin caused a mitotic delay by approximately 40% (Supplementary Fig. 2D), in accordance with a function of myosin II in contraction of the cleavage furrow during cytokinesis [38, 39]. Together, Y-27632 and blebbistatin are capable inhibitors of actomyosin contraction for functional migration experiments.

**Fig. 7:**
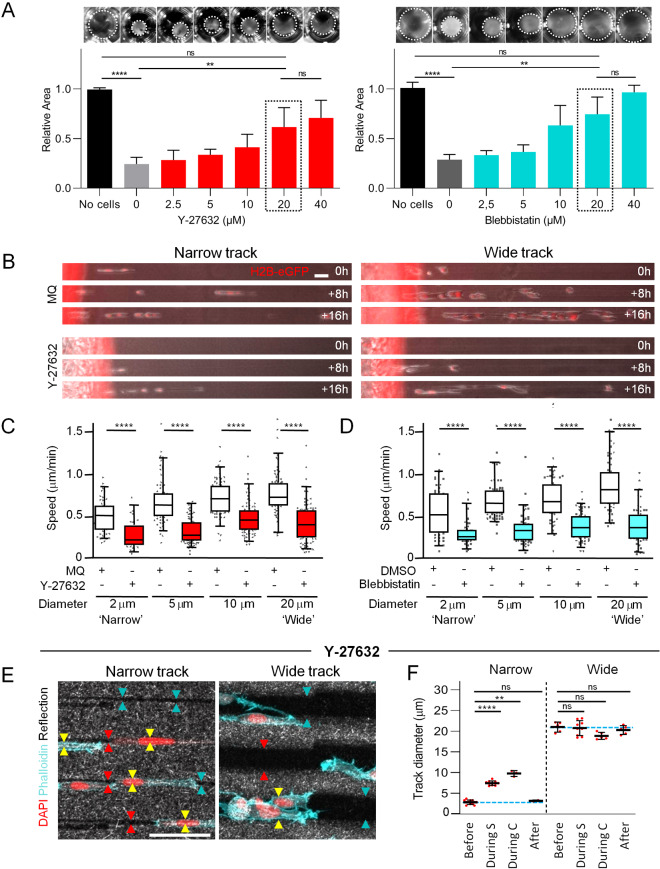
Dependence of fibrosarcoma cell migration on actomyosin contractility through microtracks of all diameters. **A** Dose-dependent inhibition of collagen gel contraction (top; dotted circles indicate collagen area) and quantification (bottom). Collagen areas were related to normalized control gel without cells. Bars and error bars, mean and SD. Data represent 7-9 gels per condition (*N* = 3). **B** Representative image series depicting cell migration through microtracks of indicated width in the absence or presence of Y-27632. **C**, **D** Mean migration speed of individual cells at indicated conditions over 6–22 hours. Horizontal lines and boxes and whiskers show the medians, 25th/75th, and 5th/95th percentile. Dots show all individual measurements outside of the 25th to 75th percentile. Data represent 47–80 cells per condition (*N* = 3–4). **E** Migrating cells in tracks of indicated diameter in the presence of Y-27632. Arrowheads, visualization of track boundaries before (cyan), during (yellow) and after (red) passage of the cell body; note the equal distance between arrowheads along the track length. **F** Track widths before, during (‘During S’, during single cell transmigration; ‘During C’, during collective cell transmigration) or after cell passage of narrow or wide tracks in the presence of Y-27632. Data represent 3–12 measurements (dots) per condition (*N* = 1). Solid line with whiskers, mean and SD. **A**, **C**, **D** Kruskal-Wallis test with Dunn’s multiple comparisons test; **F** Mann-Whitney test. Ns, not significant; **, *p*-value $< 0.01$; ****, *p*-value $< 0.0001$. Bars: 50 $\mu$m

When challenging the ability of cell contraction, we noticed that cell entry into the tracks was somewhat delayed, especially in narrow tracks, yet cells migrated in all track sizes, although generally in lower numbers compared to the control condition (Fig. 4B; Supplementary Movies 3 and 4). After track entry, the migration speed of all contraction-compromised cells was reduced in tracks of all diameters compared to control conditions (Fig. 4C, D). This reduction, when calculating the median speed of individual cells from each condition, ranged from 44-64% of the original migration of untreated cells. When considering the migratory morphology, we noted that treated cells were characterized by long cellular protrusions, yet migrated by a combination of single-cell, strand-like or clustered mode (Fig. 4B). Thus, after near-abrogation of contractility migration persists, with a speed reduction to around half in all channel sizes, as compared to 90% migration inhibition in collagen networks [22]. We finally studied the impact of contractility on cell-mediated deformation of both wide and narrow ECM tracks. Upon treatment with Y-27632, the diameter of migrating cells was, as in control cells, larger than the initial narrow track diameters (Fig. 4E). As a result, narrow track diameters increased by a factor 2–5 around the cell body, but returned to their initial diameter after cell passage (Fig. 4E, F). Accordingly, wide track walls were not deformed during the passage of Y-27632-treated cells, and diameters remained unchanged afterward, indicating that collagen deformation was lost. Thus, contractile cell–matrix interaction is required to deform the cell–collagen interface and to cause persistent track widening and narrowing, respectively.

**Fig. 8:**
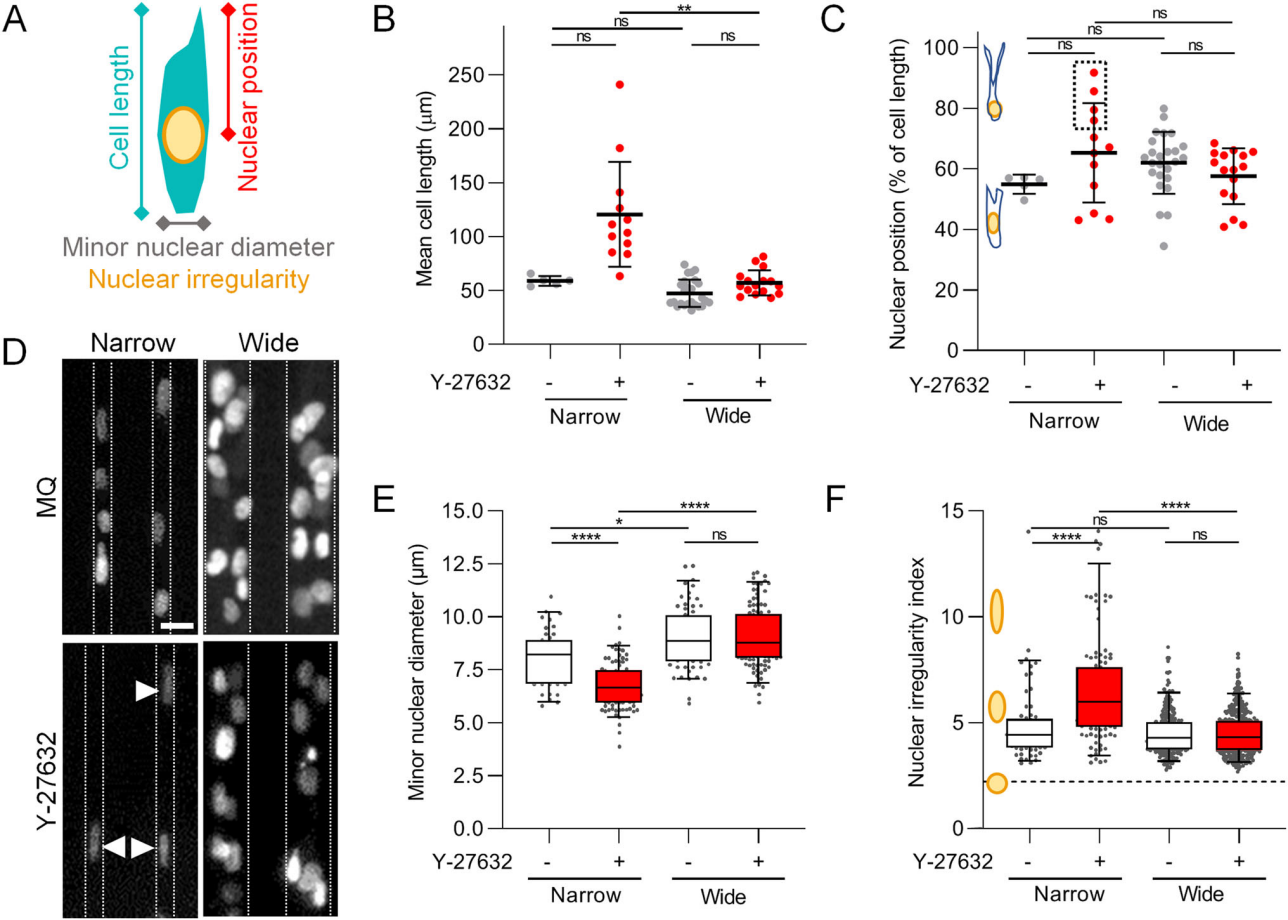
Cell and nuclear morphology in microtracks in the absence or presence of Y-27632. **A** Schematics depicting cellular and nuclear parameters analyzed. **B** Quantification of mean cell length over time at indicated conditions. **C** Quantification of mean nuclear position over time calculated as percentage of cell length over time. Dotted box indicates cells in which the nucleus located at cell rear, as indicated in the drawing. **(B,C)** Data represent mean value of 5-25 cells (dots) per condition over 3-13 hours (N=1). Solid line with whiskers, mean and SD. **D** Image of fluorescent nuclear H2B-mCherry signal at indicated conditions. Dotted lines indicate track walls, arrowheads indicate strongly deformed nuclei. **E**, **F** Quantification of minor nuclear diameter (**E**) or nuclear irregularity index (**F**) at indicated conditions. Horizontal lines and boxes and whiskers show the medians, 25th/75th, and 5th/95th percentile. Dots show individual measurements outside of the 25th to 75th percentile. Data represent 42-105 nuclei per condition (**E**), or 63-439 nuclei per condition, each from 2 experiments (**F**). **B**, **C**, **E**, **F** Kruskal-Wallis test with Dunn’s multiple comparisons test; ns, not significant; *, *p*-value $< 0.05$; **, *p*-value $< 0.01$; ****, *p*-value $< 0.0001$. Scale bar: 20 $\mu$m

**Nuclear deformation upon ROCK-inter-ference in narrow tracks**. We reasoned that two different mechanisms accounted for the reduced migration speed in both conditions, namely a pulling deficit in wide channels, versus insufficient propagation of the stiff and large nucleus in narrow tracks. We therefore studied the impact of contractility on cellular and nuclear morphology, and nuclear position and shape during migration (Fig. 5A). Upon ROCK-inhibition, all these parameters were unchanged in wide tracks, whereas multiple cellular and nuclear morphological changes occurred in narrow tracks (Fig. 5B–F). Interference with contraction in cells migrating in narrow tracks resulted in a highly heterogeneous increase in cell length (Fig. 5B). Although the position of the nucleus within cells was not altered for the majority of ROCK-inhibited cells, a subpopulation of these cells in narrow tracks had a tendency to drag the nucleus at its rear-end for prolonged times (Fig. 5C), consistent with the notion that the actin cytoskeleton positions the nucleus within the cell [40, 41]. When assessing the shape of the nucleus at the different conditions, we observed that minor nuclear diameters narrowed upon migration in confinment. Interference with ROCK decreased the nuclear diameter even further (Fig. 5D, E), consistent with findings that cells become softer after inhibition by cell contractility [42]. Accordingly, nuclear shapes were more irregular in narrow tracks in ROCK-inhibited cells compared to non-treated cells, reflecting decreased nuclear width and increased nuclear length (Fig. 5F). Thus, an active Rho/ROCK pathway is indispensable for cells in narrow tracks to maintain cellular and nuclear morphology for effective migration.

## Discussion

We here show that barrier-free deformable collagen-based microtracks with high or low level of confinement support mesenchymal fibrosarcoma cell migration in dependence of actomyosin contractility. The data further show that cells apply forces in the nN range to generate stress or strain to deform the stubstrate and that, vice versa, substrate geometry and spacing impact cell shape. Thus, efficient migration in microtracks is supported by reciprocal cell–tissue deformation to achieve physical space accommodation and is suggested to be impacted by the stiffness of both the cell and its environment.

**Space negotiation in confining environments**

During movement within complex collagen-based 3D substrates, the morphology of a polarized mesenchymal proteolytic cell converts into an elongated shape of around 10 $\upmu$m in diameter and 50-100 $\upmu$m$^{2}$ in cross section, respectively [22]. When tissue space is smaller than this cell size, i.e., in narrow tracks, the moving cell has to negotiate the available space by either (i) adapting its shape to match the degree of confinement or (ii) by generating space [32, 43, 44]. To adapt its shape, a cell needs to deform its cell body including the stiff nucleus that has been identified as the rate-limiting organelle: if tissue size is smaller than the nucleus can maximally deform the cell will undergo migration arrest [22, 44, 45]. Here, we show that cells in narrow, when compared to wide, tracks only modestly reduced migration speed and nuclear diameter, consistent with the notion that the cell nucleus forms elongated, but not the complex hour-glass, morphologies typical for migration in confining networks [22]. This may contribute to the largely unimpeded and relatively higher migration efficacies in narrow-continuous as compared to fibrillar-discontinuous spaces. Further, moving cells may negotiate confinement by increasing space, either (iia) by MMP-mediated substrate degradation leaving behind a proteolytic path exactly matching the cell size [22, 33, 34] or (iib) exerting pushing forces onto the pliable ECM substrate [42]. Consistent with these concepts, we observed that when microtracks are transmigrated by proteolytic cells, this will lead to a degradation signal linearly localized along the track wall, and over time to loss of track-mediated guidance, shown by both small and massive outbreaks of cells. We assume that this reduced collagen integrity will lead to lowered stiffness at the cell–collagen interface, consistent with ECM degradation-derived local softening at invasive fronts of collagen-embedded mammary epithelial EpH4 cell clusters [46]. To focus on the physical deformation of intact ECM with defined stiffness, but not protease-dependent ECM plasticity, we here minimized proteolytic collagen degradation by a broad-spectrum MMP-inhibitor. Consequently, in accordance with data shown by Ilina et al [32], during migration in the most confining tracks cells widened the narrow track diameters up to tenfold by physical means. Similarly, widening of pre-existing tissue tracks has also been observed *in vivo*, where during perimuscular invasion tumor cells widened narrow linear spaces by twofold to 5-10 $\upmu$ m diameter [7]. Our data presented here are further in accordance with a model established in Zanotelli *et al*. [42]: ECM confinement induces cell deformation, and the presence of the cell, especially the stiff nucleus, provides resistance and indents the ECM. These interactions concurrently deform both the cell including its nucleus and the ECM until a reciprocal equilibrium is reached and the path size matches the cell dimension. This interconnected dual deformation enables migraion in tracks initially smaller than the cell’s size without the need for MMP-mediated ECM remodeling.

**The impact of cell or substrate stiffness in actomyosin contractility-dependent space negotiation**

The generation of actomyosin-related force necessary for ECM deformation costs energy, which increases with ECM confinement and cell- and ECM stiffness [47]. We here relate actomyosin contractility to its function to provide intra- and extracellular forces that contribute to high-speed migration in linear deformable ECM tracks. First, engagement of the nucleus by the actomyosin network and tension-mediated regulation of lamin A/C expression maintains nuclear stiffness, and upon interference with actomyosin contractility cell stiffness decreases [42, 48–51]. Second, actomyosin contraction is required to transport the nucleus through small spaces [15, 22] that, when inhibited, results in rear-oriented nuclear positioning as was observed in a subset of our cells migrating in the narrow tracks. Third, cells exert contractile traction forces toward collagen thereby pulling on and deforming the environment. The estimated traction forces of up to 40 nN onto collagen by by 2D speckle tracking are consistent with findings on approximately 30 nN-strong traction forces by mouse embryonic fibroblasts within collagen analyzed by 3D particle velocimetry, that in vinculin KO cells reduced to traction forces less than 1 nN [52]. Fourth, actomyosin contractility associated with persistent widening of narrow tracks is indicative of lateral forces including nuclear stiffness. Consequently, the use of Y-27632 inhibitor led to a reduction of lateral forces applied upon the substrate associated by a decrease in nuclear diameter indicative of reduced nuclear stiffness. Thus, although actomyosin contractility-derived force is not required for the principal capacity of cells to migrate in confining tracks, it regulates cell stiffness, nuclear positioning and matrix traction and hereby provides the necessary force to enhance ECM deformation, allowing for efficient migration.

Substrate stiffness represents a counter-force to cell stiffness, and therefore, the rigidness of the environment invaded by tumors needs to be considered. The stiffness of the collagen used in this study was relatively low (0.34 kPa), whereas loose fibrillar collagen type-I- and type-III-based porous protein networks, such as in mouse dermis and breast *in vivo*, scale typically between 0.2 and 3 kPa [reviewed in van Helvert, 2018 [5]]. In progressing MMTV mouse tumors, the elastic modulus of mammary glands increases from 0.8 to 1.7 kPa [53], and skeletal myofibers are with around 12 kPa even stiffer [3, 54]. Thus, whereas the often used synthetic PDMS migration channels of 1000 kPa stiffness [31, 55] are much above the stiffness of *in vivo* tissues typically invaded by tumors, the collagen hydrogels used here scale in their soft range. However, even though our microtrack model represents a relatively soft environment, it has been shown that invading cells can widen linear track-like spaces surrounded by environments of higher stiffness as well. For example, Zanotelli and colleagues treated a 3 mg/ml collagen hydrogel with ribose, which increased the stiffness from 0.4 to 0.55 kPa [42, 56]. In this setting, single migrating cells kept deforming the 7 $\upmu$m-wide tracks to the same extent (by ~1.5 $\upmu$m), albeit at the cost of higher glucose uptake. Thus, migration-associated track widening in confining tracks is an energy-consuming process where ATP consumption in the cell increases the stiffer the ECM is [42] but, as long as sufficient energy is present, takes place. In line with these findings, melanoma cells migrating in tracks between myofibers in a live mouse were able to open confined track space by around 2 times from around 3 to 5-10 $\upmu$m [7]. From this data, we conclude that ECM deformation behavior of linear confinement is relevant for both softer or stiffer environments.

What is then the requirement of actomyosin contractility for migration in linear confinement of different stiffness? Cell migration through confining non-deformable PDMS-based tracks did not depend on Rho/ROCK-dependent cellular contractility [25], and a compensatory, osmosis-related mechanism has been proposed for the ongoing migration activity of cells devoid of myosin activity [57]. In our deformable collagen tracks, in contrast, cell speeds were reduced by approximately 35-55% upon ROCK or myosin II interference, regardless of channel cross section. It will be therefore interesting to investigate what the dependence on actomyosin contractility is for migration in track-like topologies of physiologic environments that better match the medium stiffness ranges of human tissues.

**Outlook**

In summary, we have shown that migrating mesenchymal cancer cells morphologically adapt to the geometry of their microenvironment, but reciprocally also deform the environment by actomyosin contractility to create an optimal balance between available substrate space and cell deformation needed for optimized migration. Further studies should investigate the behavior of different, i.e., epithelial, cancer types of varying epithelial–mesenchymal transition states in microtrack migration, as well as further integrate the various topologies, space availabilities and stiffness ranges of invaded tissues *in vivo * [5]. Lastly, the relevance of the tumor invasion delay induced by targeting of actomyosin contractility observed here requires further studies in the context of anticancer invasion and metastasis treatment in the living organism *in vivo*.

## Supplementary Information

Below is the link to the electronic supplementary material.

Supplementary file 1 (avi 4639 KB)

Supplementary file 2 (avi 1305 KB)

Supplementary file 3 (avi 14381 KB)

Supplementary file 4 (avi 11533 KB)

Supplementary file 5 (pdf 596 KB)
