# Supplementary material for: Actomyosin contractility requirements and reciprocal cell–tissue mechanics for cancer cell invasion through collagen-based channels
Source: Eur Phys J E Soft Matter. 2022 May 16;45(5):48. doi: 10.1140/epje/s10189-022-00182-6 (PMC9110550; doi:10.1140/epje/s10189-022-00182-6)
Supplement: Supplementary file 5 — (pdf 596 KB) [file 10189_2022_182_MOESM5_ESM.pdf]

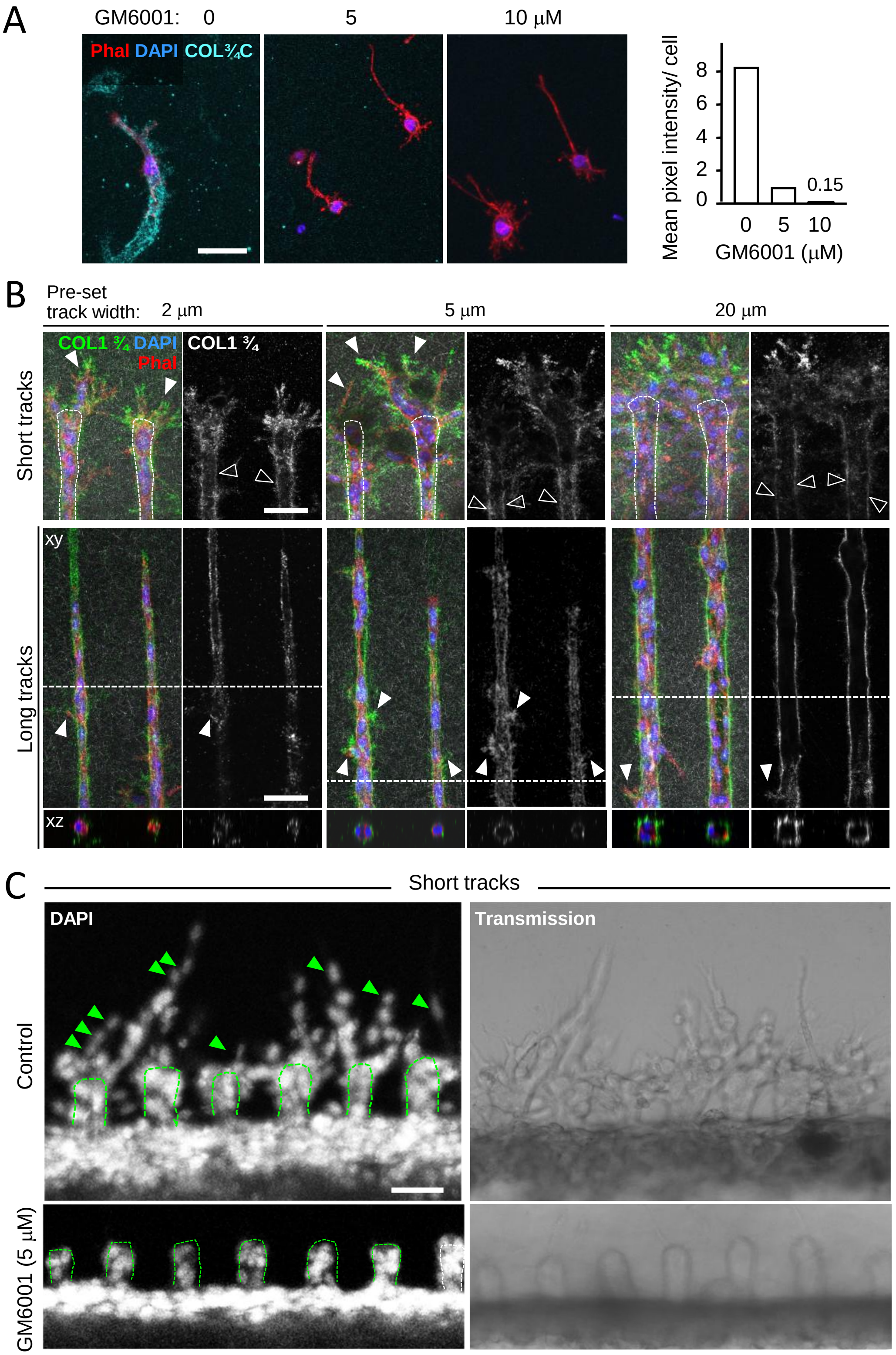

Supplementary Figure 1. Impact of tumor cell proteolysis on migration in collagen models. HT1080 cells migrated in 3D collagen **(A)** or collagen-based microtracks (**B,C**) for 24 hours before fixation. **(A)** Imaging (left) and quantification (right) of efficacy of broad-spectrum MMP inhibitor GM6001 on cell-derieved collagen degradation. N=1; each 5-7 cells per condition. **(B)** Visualization of cell migration inside and outside tracks and related collagen degradation. Top row, short tracks, indicated by dotted lines. White arrowheads, massive breakout of cells at the end of the tracks; empty arroheads indicate collagen degradation signal along tracks. Lower row, xy and xz views of long linear tracks. Dotted horizontal lines indicate position of xz view generation. White arrowheads, focalized signal where cells probe collagen outside of the confining tracks. **(C)** Breakout of cells (arrowheads) from wide short tracks (20 x 40 μm; dotted lines) is inhibited in the presence of GM6001. All bars, 50 μm.

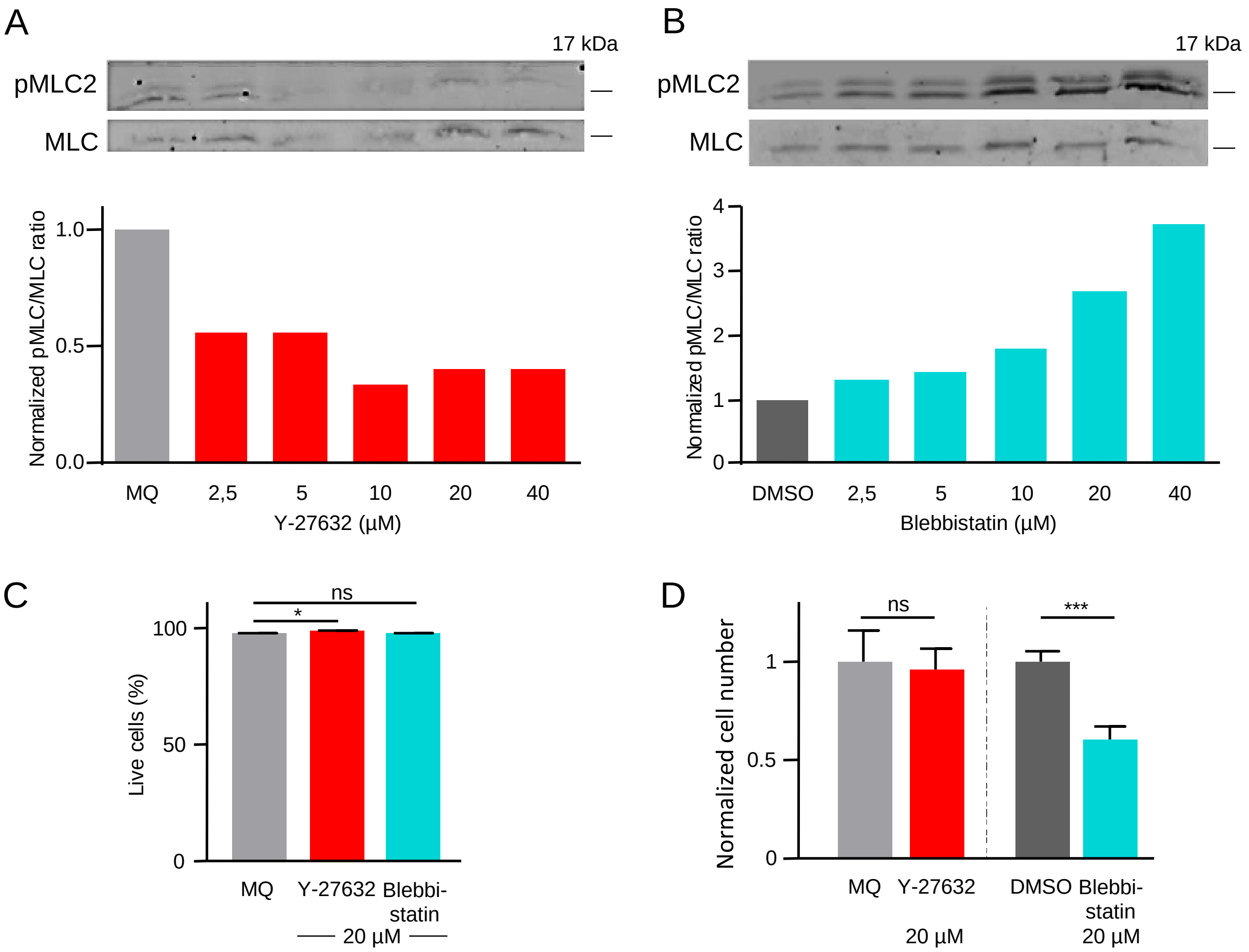

Supplementary Figure 2. Dose-dependent effects of Y-27632 and blebbistatin on MLC phosphorylation and cell function. Cells were treated with indicated concentrations of Y-27632 and blebbistatin for 1 hour before being lysed for western blotting **(A,B)** or for 24 hours before being prepared for the respective assays **(C,D)**. **(A,B)** Western blot images of phosphorylated myosin light chain 2 (pMLC2) and MLC protein expression (top) and respective quantification by densitometry and shown as normalized ratio (bottom) after indicated treatments; data represent 1 measurement per condition. **(C)** Confirmation of uncompromised cell viability in the presence of indicated inhibitors, using propidium iodide FACS assay to determine toxicity. Depicted is the percentage of propidium iodide-negative cells. Data represent 3-6 wells per condition (N=1). **(D)** Cell proliferation in the absence and presence of indicated inhibitors after 24 hour-treatment. Individual results were normalized to the mean cell count of the corresponding control sample per experiment. Data represent 9 wells per condition (N=3). **(C,D)** Bars and error bars, mean and SD. **(C,D)** Kruskal-Wallis test with Dunn's multiple comparisons test; ns, not significant; *, p-value < 0.05; ***, p-value < 0.001.

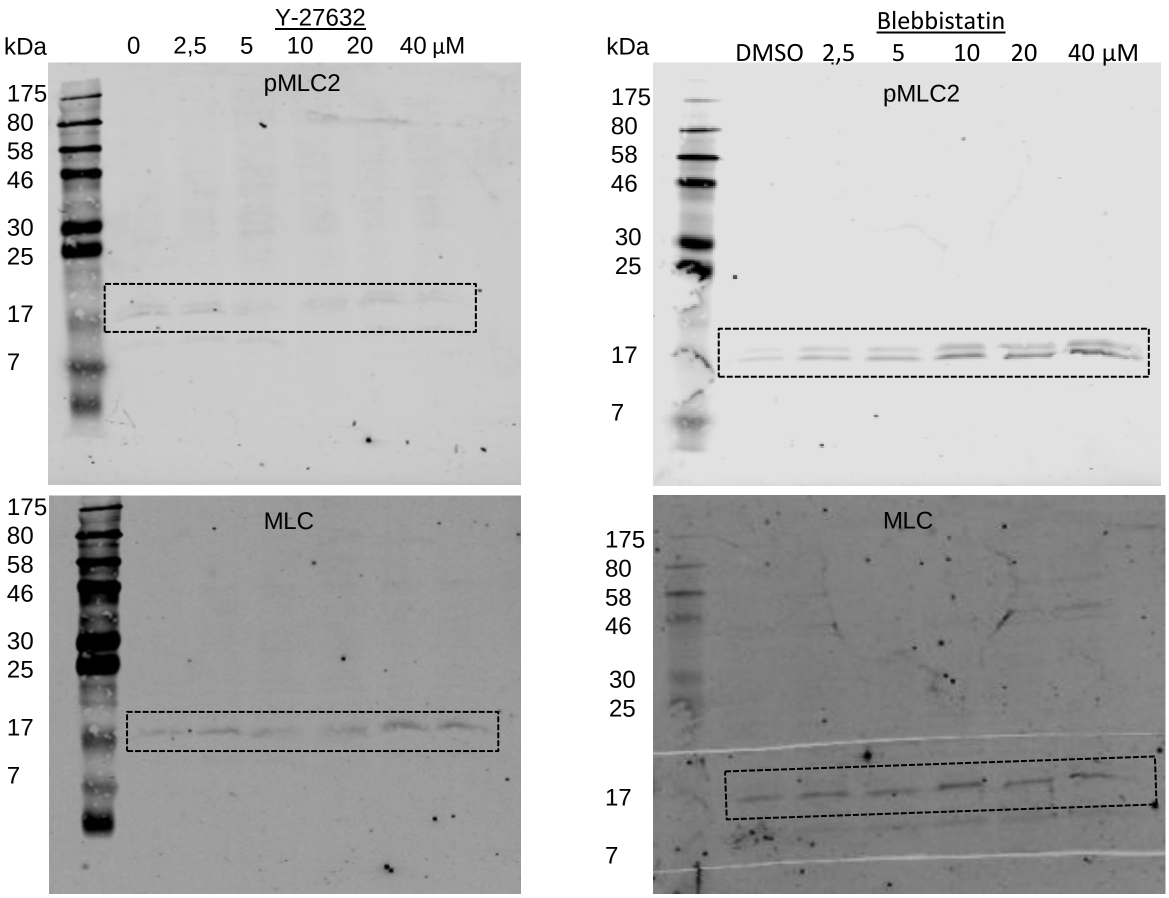

Supplementary Figure 3. Original Western blots. Dotted rectangles correspond to processed cropped images in Fig. S2A and B.
